# Inclusion of HPV testing in routine cervical cancer screening for women above 29 years in Germany: results for 8466 patients

**DOI:** 10.1038/sj.bjc.6600918

**Published:** 2003-05-13

**Authors:** K-U Petry, S Menton, M Menton, F van Loenen-Frosch, H de Carvalho Gomes, B Holz, B Schopp, S Garbrecht-Buettner, P Davies, G Boehmer, E van den Akker, T Iftner

**Affiliations:** 1 Abteilung für gynäkologische Onkologie MHH, Hannover, Germany; 2 Frauenklinik, UKT, Tuebingen, Germany; 3 Department of Public Health, Erasmus University, Rotterdam, Netherlands; 4 Experimentelle Virologie, UKT, Tuebingen, Germany; 5 ECCCE, London, UK

**Keywords:** cervical cancer screening, HPV test performance, cytology performance

## Abstract

In a prospective cohort study 8466 women attending routine cervical cancer screening were recruited. Colposcopy was performed on women with any degree of atypia on cytology and/or a positive high-risk human papillomavirus (HPV)-DNA test (HC2; Hybrid Capture 2^©^), and for a randomly selected sample of 3.4% women with negative findings on both. Quality control included reviews of cytology, histology, colposcopy images and retesting of samples with polymerase chain reaction. Test diagnostic performances were based on 7908 women who had complete baseline and follow-up results. Routine histology identified 86 women with high-grade cervical intraepithelial neoplasia (CIN2+), which was confirmed by review histology in only 46 cases. Sensitivity of routine cytology for the detection of CIN2+ was 43.5%, with a specificity, positive predictive value (PPV), negative predictive value (NPV) of 98.0, 11.4 and 99.7%, respectively. Sensitivity of the HC2 test for the detection of CIN2+ was 97.8%, with a specificity, PPV and NPV, of 95.3, 10.9 and 100%, respectively. No high-grade neoplasia was detected in the randomly selected control group. A negative HPV-test result, even in combination with a positive Papanicolaou (Pap) result, virtually excluded any risk of underlying high-grade disease, but this was not the case for a negative Pap result. These data show that HPV testing is of value for the detection or exclusion of prevalent CIN in a routine cervical cancer-screening setting and could be used for further risk classification of women for follow-up management.

Despite widespread cervical cancer-screening programmes, 30 000 of the 190 million women living in Europe still die every year from this disease (Ferlay *et al*, 1988). The overall decline in cervical cancer incidence observed since the introduction of the Papanicolaou (PAP) smear has levelled off and even started to increase in countries such as England, Wales and Finland (Vizcaino *et al*, 2000), where organised screening programmes with extensive quality control have been in place for many years.

In Germany, an opportunistic cervical cancer-screening programme was established in 1971, which made free annual cervical cancer screening available to all women 20 years of age or older. The average annual participation rate in 1997 was roughly 50% (Kahl *et al*, 1999; Klug *et al*, 2000; Klug and Blettner, 2003). When looked at over a 3-year period, which is the screening interval used in many European countries, the population coverage reaches 80% (Schenck and von Karsa, 2000) or higher in the 25–50-year-old age group. Despite this considerable effort, Germany has one of the highest cervical cancer incidence rates (13.28 per 100.000) and standardised mortality rates (4.4 per 100.000 for 1993) in Western Europe, which is higher than in neighbouring countries with similar socio-economic structures such as France, Italy and the Netherlands.

Infection with high-risk human papillomavirus (HR-HPV) is the major risk factor for the development of cervical cancer (Bosch *et al*, 1995; Walboomers *et al*, 1999) and it has been suggested that testing for HR-HPV should be included in existing cervical cancer-screening programmes (Vizcaino *et al*, 2000). Large-scale screening studies by Schiffman *et al* (2000), Clavel *et al* (2001) and Schneider *et al* (1996),(2000) demonstrated that HPV testing is more sensitive for the detection of high-grade cervical lesions than cytology although the lower specificity of HPV testing made its use in primary cervical cancer screening questionable. However, the majority of these studies did not compare HPV testing to routine cytology, but rather used a team of expert cytologists thereby not allowing to draw conclusions about the performance of HPV testing under routine screening conditions. In addition, these studies included a high proportion of younger women aged 18 years and above, although it is known that young, sexually active women have a high prevalence of both HR-HPV and high-grade cervical intraepithelial neoplasia (CIN), the overwhelming majority of which resolves spontaneously (Evander *et al*, 1995; Ho *et al*, 1995). It is possible that the higher sensitivity of HR-HPV testing in these young populations in comparison with cytology may partially be because of the identification of transient disease with a high tendency for regression. To address this particular issue, Cuzick *et al* (1999) examined the performance of HPV testing in women 35 years of age or older and again found that HR-HPV testing had a higher sensitivity than cytology, but a lower specificity and positive predictive value (PPV) leading to a higher referral rate than cytology alone. Importantly, all of the former studies are characterised by the lack of an HPV−/Pap− control group examined by colposcopy, thereby not allowing an accurate estimate of the false-negative rate of the screening regimen (Ratnam *et al*, 2000).

Our aim was to determine the value of HPV testing in the routine primary cervical cancer-screening programme in Germany for the detection of high-grade cervical cancer precursors (⩾CIN2). To avoid confounding factors, we implemented different levels of quality controls (see study protocol in Figure 1

**Figure 1:**
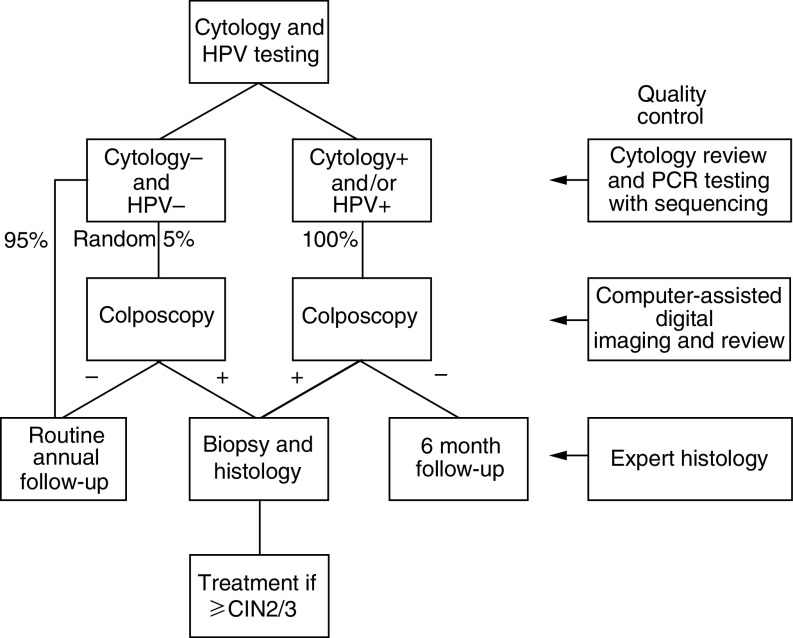
Study Protocol.

) to evaluate the use of HPV testing. All the 8083 study participants finally included were aged 30 years or older attending for routine cancer screening and represented a random unselected population that was highly representative of Western Germany as a whole.

All positive and 5% of the negative HC2 samples were retested by a highly sensitive, broad-range, consensus-primer PCR with direct sequencing to evaluate HC2 performance. Digital images of the cervix were taken at the colposcopic examination and reviewed by independent experts blinded to previous findings. Additional expert reviews were performed on all histology samples and all cytology samples with any degree of dyskaryosis, all cytology samples from HR-HPV+ women and 5% of randomly selected cyto-/HPV-cytology samples. Most importantly, a colposcopic examination of a random sample of 250 women who were cytology and HPV− was performed in order to estimate the bias that would be introduced by disease missed in women who were falsely negative on both tests (Ratnam *et al*, 2000). Cytology labs undertaking the primary cytology were not informed about which Pap smears were included in the trial and the samples were therefore screened under routine conditions. The statistical analysis of the raw data was performed by an independent group (Erasmus University, Rotterdam) that was not involved in the screening trial nor in the collection of the data.

## MATERIALS AND METHODS

### Study population

Between December 1998 and December 2000, 8466 women attending routine cervical cancer screening were recruited from 28 urban, suburban or rural, office-based gynaecological practices from Hannover and Tuebingen and the surrounding areas. Women were eligible for inclusion if they were attending for routine annual cervical cancer screening, were 30 years of age or older, had not undergone a hysterectomy, had no history of atypical cytology, CIN, or treatment for cervical disease in the preceding year and were currently not pregnant.

Of the 8466 women enrolled in the study, 8101 women met the inclusion criteria. Of the 4566 participants from Tübingen, 233 (5.1%) were excluded, leaving 4333. Of the 3900 women enrolled from Hannover, 132 women (3.4%) were excluded leaving 3768. Reasons for exclusion (365 women) were: 43 women with genital warts; 13 women with a history of conisation or hysterectomy; 11 women who were pregnant; eight women with abnormal cytology within 1 year of study entry; 167 women who were under the age of 30 years and 123 women who had not given written consent.

Written informed consent was obtained from the patients by the participating gynaecologists. The study was approved by the local ethics committees at the Universities of Hannover and Tuebingen.

### Screening examinations

At the first gynaecological examination, the cervix was visualised and a sample was taken for routine cervical cytology following the procedures normally used in each gynaecological practice (most, but not all, samples were taken with a cotton-tipped swab, rolled onto a microscope slide and spray fixed). This practice of sample taking is the standard recommended procedure in Germany (Link M and Link H, 2001). A second sample was then obtained with a cervical sample device (Digene Inc. Gaithersburg, MD, USA) and suspended in 1 ml of specimen transport medium (STM/ Digene Inc, Gaithersburg, MD, USA) for HPV DNA testing. Women were allocated to follow-up according to the protocol illustrated (Figure 1).

### Cytological diagnoses

All cervical smears were analysed at one of the cytology laboratories normally used by each participating office-based gynaecology practice and reported in accordance with the Second Munich Cytological Classification (Table 1

**Table 1:** Correlation between second Munich classification and the Bethesda system

Munich classification	Bethesda correlate
PapI/II	Normal/inflammatory
PapIII	ASC-H and AGUS–cannot exclude high-grade disease or cancer
PapIIId	CIN1–2
PapIva	CIN3–carcinoma *in situ*
PapIvb	Carcinoma *in situ*–microinvasive carcinoma
PapV	Microinvasive carcinoma–invasive carcinoma
	
Unofficial classification	Bethesda correlate
PapIIw	Includes inadequate and ASC-US

). The ‘Pap II w’ (W=wiederholen=repeat) is a widely used category although it is not an official cytological classification. It is used by cytologists to describe inadequate specimens, minimal changes and koilocytes in the absence of further abnormalities or atypical squamous cells of undetermined significance (ASCUS). In this study, smears were considered positive if any degree of cytological abnormality was observed (⩾PapIIw, ≈borderline/ASCUS) in order to maximise detection of disease. The cytology laboratories had not been informed about the study and were therefore analysing the smears under routine screening conditions. All glass slides and accompanying forms lacked information on the patient's participation in a trial to prevent bias in reading.

### HPV DNA testing by the HC2 assay

Samples for HPV testing were stored at 4°C for a maximum of 4 weeks prior to testing. All primary HPV testing was undertaken using the Hybrid Capture 2 test (HC2/Digene Inc, Gaithersburg, MD, USA) at the clinical diagnostic laboratory of the Medizinische Hochschule Hannover or at the Department of Experimental Virology at the University of Tuebingen. All samples were analysed for the presence of 13 HR-HPV types (16, 18, 31, 33, 35, 39, 45, 51, 52, 56, 58, 59 and 68) following the manufacturer's instructions. Therefore, a positive HPV result refers to a patient who is positive for one or more of the 13 HR-HPV types included in the high-risk probe mix. Infection with low-risk HPV types was not evaluated. Samples were considered positive if they attained or exceeded the FDA-approved threshold of 1.0 pg HPV DNA ml^−1^, which corresponds to 1.0 relative light units (RLU). All samples with RLU values in the range of 0.7–2.0 RLU were retested in duplicate, although subsequent statistical analysis was based only on the primary measurement.

### HPV testing by PCR

DNA extraction, sample preparation and sample analysis procedures were carried out in separate rooms. All samples were tested for DNA integrity by PCR using primers for the human *β*-globin gene. Three different primer combinations were used (PPF1/PPR2, PPF1/CP5, CP4/CP5) producing amplicons ranging in size from 220 to 450 bp, all located in the highly conserved helicase region of the E1 gene. The sensitivity of this PCR system for different HPV types was established by testing serial dilutions of HPV plasmids for the following types: HPV1–8, 10–19, 21–26, 30–38, 40, 45–47 and 60, both in the presence and absence of human placenta DNA (1 *μ*g). Primers PPF1/PPR2, which are not degenerate, reach the highest sensiti-vity of 10 genome copies per sample and result in a single 220 bp amplification product when applied to DNA extracted from clinical samples. PPF1/CP5 and CP4/CP5 have a detection limit of 100 copies, which is by far sufficient for the testing of clinical samples of the cervix. Primers PPF1/CP5, where the downstream primer is degenerate in three positions, detect at least 64 different HPV types and produce a 280 bp amplicon, while primers CP4/CP5 with five degenerate nucleotide positions generate a fragment of 450 bp that has been described elsewhere (Tieben *et al*, 1994). The nucleotide sequence of the primers is as follows:

PPF1: 5′-(nt 2082)-AAC-AAT-GTG-TAG-ACA-TTA-TAA-ACG-AGC-(nt 2108)-3′
PPR2: 5′-(nt2336)-ATT-AAA-CTC-ATT-CCA-AAA-TAT-GA-(nt2314)-3′
CP4: 5′-(nt1942)-ATG-GTA-CAR-TGG-GCA-TWT-GA-(nt1961)-3′
CP5: 5′-(nt 2400)-GAG-GYT-GCA-ACC-AAA-AMT-GRC-T-(nt 2378)-3′

Numbers are according to the sequence of HPV16W12E (Genbank IDNr. AF125673).

Direct automated sequencing of the PCR products was carried out using the Big Dye Terminator Cycle Sequencing Ready Reaction Kit (PE Biosystems, Foster City, CA, USA). Sequencing was performed in 47 cm capillaries with the use of an ABI 310 sequencer (PE Biosystems). Sequences with <5% unidentifiable bases were processed and compared with those of known HPV types available through the GenBank database (NCBI, Bethesda, MD, USA) using the Blast program. A nucleotide sequence was regarded as a distinct HPV type if it demonstrated <90% homology with a known type.

### Colposcopic referral

Women were called for colposcopy at either of the two university gynaecology clinics if they demonstrated any degree of cytological abnormality (⩾PapIIw; ≈borderline/ASCUS) and/or if they were positive for HR-HPV. In addition, a random sample of 5% of women who were negative on both screening tests were invited to attend for colposcopy in order to assess the prevalence of high-grade disease in this population. The median recall interval (time from initial screening examination to attendance for colposcopy) was 79 days with a range from 12 to 128 days. At colposcopy, punch biopsy specimens were taken from any regions suspicious for CIN and final diagnoses were based on histological results expressed using the Bethesda terminology. Cases were defined as women with high-grade cervical disease (⩾CIN2) using expert histology based on the primary histological result and two independent, blinded reviews.

### Quality control procedures

An extensive series of quality control procedures was implemented (Figure 1). All abnormal cytology, all cytology from women positive for HR-HPV and 5% of normal cytology were mixed 1 : 1 with normal Pap smears and reviewed by one of the two independent expert cytopathologists (Professor Dr med. M Hilgarth, Professor Dr med. N Freudenberg, Universitätsfrauen-klinik Freiburg). All statistical analyses were based on primary cytology and review cytological diagnoses were used for quality control purposes only. All histological specimens were reviewed by an independent expert histopathologist (Professor Dr Hans-E Stegner, University of Hamburg/retired) and specimens showing significant disagreement between primary and review histology were referred to a third histopathologist (Dr C Bergeron, Laboratoire Pasteur Cerba, France). All statistical analyses were based on combined expert histology achieved in the following way:

1. All histology diagnoses were classified into cases (⩾CIN2) and noncases (<CIN2, VIN, VaIN).
2. If routine histology and the review differed between those categories, a third histology diagnosis (second review) was included. A patient was then defined as case if the quotient of case/noncase was 2.0 and as noncase if the quotient was 0.5.

All independent experts were completely blinded to previous findings. Colposcopy was quality controlled using a computer-assisted digital imaging system (DenVu, Tucson, AZ, USA) initially developed for the ASCUS-LSIL Triage Study (ALTS) conducted by the NCI (Solomon *et al*, 2001). Finally, HPV-DNA testing was quality controlled by the inclusion of three known positive samples and three known negative samples that are included with the HC2 kit; the modification of HC2 analysis software to require the inclusion of four blind samples with each test run, which must give results within a predetermined range for the run to be valid (blind control samples provided by Digene Inc). In addition, 7.5% of all HC2 samples were exchanged between Hannover and Tuebingen for retesting. Both labs were blinded to previous results. In addition, all samples with either a positive test result from cytology or HC2 or from both and 5% of the negative samples were retested by PCR and positives were directly sequenced to confirm their status.

### Data analysis

All statistical analyses were undertaken by an independent group that did not participate in the screening trial or in the collection of the data. All test results were stored in a Microsoft Access database in Hannover and Tuebingen and later combined into one file which was imported into SPSS 9.0 for analysis. The cytology results were categorised into positive (⩾PapIIw; ≈borderline/ASCUS) or negative results. An HPV-HC2 result was considered positive when it attained or exceeded the FDA-approved threshold of 1.0 pg HPV DNA ml^−1^, which corresponds to 1.0 RLU. The cytology and HPV-HC2 test reviews were not used for the calculation of the test properties. Histology results were considered positive for CIN2 and higher. Vaginal intraepithelial neoplasia (VaIN) and vulvar intraepithelial neoplasia (VIN) were not considered as positive results for this analysis. The test sensitivity, specificity, PPV and negative predictive value (NPV) were calculated by contingency table analysis with 95% confidence intervals assessed using logistic regression, using a weighting factor. The women were divided into four groups according to their cytology and HPV-test results: both cytology and HPV−; cytology+, HPV−; cytology negative, HPV+; both cytology and HPV+. In women with both cytology and HPV− test results, only a small percentage has had a colposcopic examination and histology results were available. The assumption was made that this selected group of women who underwent colposcopy are representative of all the women in the double-negative group. By multiplying the cases (women with ‘final histology’ ⩾CIN2) of the double-negative group by the weight factor (total number of double-negative women divided by the number of women of this group that underwent colposcopy) the total number of cases could be estimated as if every woman would have had histology. The confidence intervals were calculated with logistic regression taking into account this weight factor. In some subgroups, test properties were 100%. In these instances, the log likelihood for the underlimit of the confidence intervals was calculated by the following formula 1−e^ln(0.05)/^*^n^*, with *n* being the number of observations in women who had a colposcopy result.

## RESULTS

The mean age of the 8101 women included in the study was 42. 7 years, with 94.6% of the women between 30 and 60 years of age. Of the 8101 women included in the study, 18 women did not have a cytology result, leaving 8083 women with both a cytology result and an HPV-HC2 result at baseline. Of the 8083 women with complete baseline results, 251 women (3.1%) had cytological abnormalities⩾PapIIw (≈borderline/ASCUS), which included one woman with PapIVb (⩾CIN3), eight women with PapIVa (CIN3), 61 women with PapIIID (CIN1–CIN2), 14 women with PapIII (ASC-H/AGUS) and 167 women with PapIIw (≈borderline/ASCUS). In all, 521 (6.4%) women were positive for HR-HPV (Table 2

**Table 2:** Characteristics of study population

	PapI+II			PapIIw			PapIII			PapIIId			PapIV+V			Total		
	*N*	%Row		*N*	%Row		*N*	%Row		*N*	%Row		*N*	%Row		*N*		
Total	7832	96.9%	%Column	167	2.1%	%Column	14	0.2%	%Column	61	0.8%	%Column	9	0.1%	%Column	8083	%Row	%Column
*Age at diagnosis*																		
30–40	3565	97.2%	45.5%	65	1.8%	38.9%	4	0.1%	25.0%	29	0.8%	47.5%	6	0.2%	66.7%	3669	100.0%	45.4%
40–50	2592	96.6%	33.1%	63	2.3%	37.7%	7	0.3%	43.0%	18	0.7%	29.5%	2	0.1%	22.2%	2682	100.0%	33.2%
50–60	1180	96.6%	15.1%	28	2.3%	16.8%	2	0.2%	12.5%	11	0.9%	18.0%	0	0.0%	0.0%	1221	100.0%	15.1%
60+	495	96.9%	6.3%	11	2.2%	6.6%	3	0.6%	18.8%	3	0.6%	4.9%	1	0.2%	11.1%	511	100.0%	6.3%
																		
Total column	7832	96.9%	100.0%	167	2.1%	100.0%	16	0.2%	100.0%	61	0.8%	100.0%	9	0.1%	100.0%	8083	100.0%	100.0%
																		
*Region*																		
Northern Germany	3639	96.8%	46.5%	89	2.4%	53.3%	4	0.1%	28.6%	23	0.6%	37.7%	5	0.1%	55.6%	3760	100.0%	46.5%
Southern Germany	4193	97.0%	53.5%	78	1.8%	46.7%	10	0.2%	71.4%	38	0.9%	62.3%	4	0.1%	44.4%	4323	100.0%	53.5%
																		
Total column	7832	96.9%	100.0%	167	2.1%	100.0%	14	0.2%	100.0%	61	0.8%	100.0%	9	0.1%	100.0%	8083	100.0%	100.0%
																		
*HPV*																		
HC2-HR negative (<1.0)	7372	97.5%	94.1%	149	2.0%	89.2%	11	0.1%	78.6%	30	0.4%	49.2%	0	0.0%	0.0%	7562	100.0%	93.6%
HC2-HR positive	460	88.3%	5.9%	18	3.5%	10.8%	3	0.6%	21.4%	31	6.0%	50.8%	9	1.7%	100.0%	521	100.0%	6.4%
																		
Total column	7832	96.9%	100.0%	167	2.1%	100.0%	14	0.2%	100.0%	61	0.8%	100.0%	9	0.1%	100.0%	8083	100.0%	100.0%

). All 711 women with a positive result for at least one of the tests were invited for colposcopy, but 175 of them refused examination. They were excluded from further analyses, leaving 7908 women meeting the inclusion criteria and having results for both tests at baseline and follow-up with 3683 from Hannover and 4225 from Tuebingen.

In all, 5% of the double-negative women were randomly selected and invited for colposcopy, 250 women (3.4%) responded to this invitation. The results for these women were considered representative for all double-negative women. Based on extrapolation of the primary histology results of the double-negative control group (five cases of CIN2+ in 250 women), we calculated that 247 out of the total cohort of 7908 women would have had the routine diagnosis of high-grade cervical disease (⩾CIN2). However, independent blinded reviews by two pathologists of all histology slides stated that none of these controls showed any degree of neoplasia. Further, review of the digital colposcopic images confirmed the absence of disease in this group. There was complete agreement between primary and review colposcopy in terms of normal colposcopy results for all those women with normal Pap smears and negative HPV tests who had no cervical biopsy.

**Figure 2:**
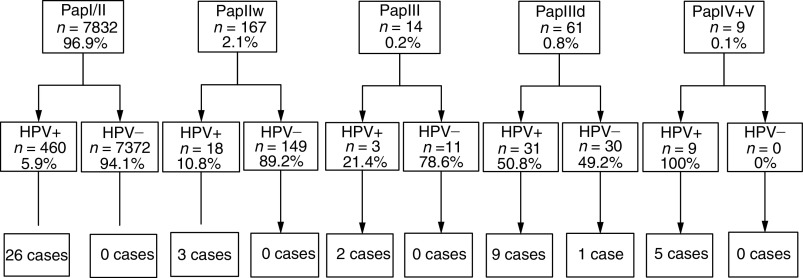
Patients grouped by cytology, HPV-test results and their confirmed histology results.

Overall, 86 cases were found on primary histology among the 536 women attending colposcopy, from which only 46 cases were confirmed on expert review histology. Of the 46 women with high-grade cervical disease on expert histology, 20 would have been detected on the basis of cytology alone (Figure 2

) using the lowest possible threshold (⩾Pap IIw) for referral to colposcopy, for a sensitivity, specificity, PPV and NPV of 43.5, 98.0, 11.4 and 99.7%, respectively (Table 3

**Table 3:** Test performance of HC2 and cytology at different thresholds for detection of histologically confirmed CIN2+ or CIN3+

	Sensitivity %		Specificity %		PPV %		NPV %		
For CIN2+		95% CI		95% CI		95% CI		95% CI	Referred
PapIIw+	43.5%	30.0–58.0	98.0%	96.7–98.8	11.4%	7.5–16.9	99.7%	98.7–99.9	2.2%
PapIII+	37.0%	24.4–51.6	99.4%	98.5–99.8	27.0%	17.5–39.2	99.6%	98.8–99.9	0.8%
HPV	97.8%	86.3–99.7	95.3%	93.5–96.6	10.9%	8.2–14.2	100.0%	55.3–100	5.2%
HPV/PapIIw+	100.0%	93.7–100	93.8%	91.8–95.3	8.6%	6.5–11.3	100.0%	98.8–100	6.8%
									
*For CIN3+*									
PapIIw+	46%	30.8–61.9	98.0%	96.7–98.8	9.7%	6.1–15	99.7%	98.8–99.9	2.2%
PapIII+	40.5%	26.1–56.8	99.4%	98.5–99.8	23.8%	14.9–35.8	99.7%	98.9–99.9	0.8%
HPV	97.3%	83.2–99.6	95.2%	93.4–96.5	8.7%	6.3–11.8	100.0%	55.3–100	5.2%
HPV/PapIII+	100.0%	93.7–100	94.9%	93.1–96.2	8.4%	6.2–11.4	100.0%	99.1–100	5.6%

).

**Figure 3:**
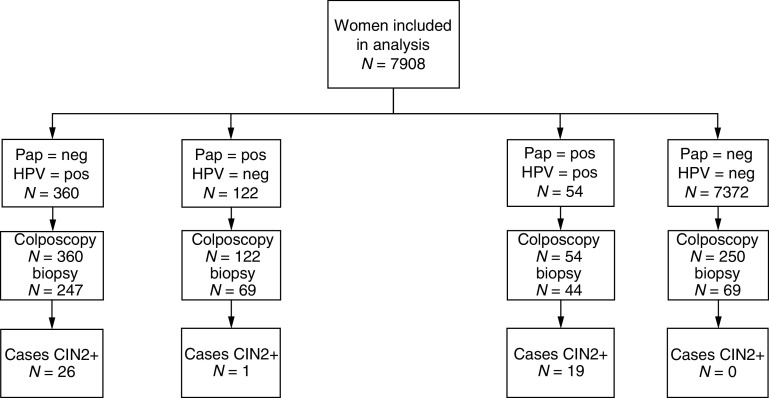
Patients divided into four groups according to their cytology and HPV test, follow-up management and confirmed histology results.

Human papillomavirus-positive status was strongly associated with the detection of cervical disease. In all, 45 of the 46 cases were positive for HPV (Figure 3

) using the 1.0 pg HPV-DNA ml^−1^ threshold, giving a sensitivity and specificity of 97.8 and 95.3%, respectively. The positive and negative predictive values of HPV testing were 10.9 and 100%, respectively. Combining cytology with HPV testing increased sensitivity to 100%, with a specificity, PPV and NPV for the combined test protocol of 93.8, 8.6 and 100%, respectively (Table 3). While this approach offered the best in terms of safety, it led to the highest number of follow-up examinations (6.8%).

Of the 37 women with CIN3 or invasive cervical carcinoma, 20 had completely normal Pap smears. Of the remaining 17 women, two had smears classified as PapIIw (ASUCS, borderline), one had PapIII (ASC-H/AGUS), nine had PapIIID (CIN1-CIN2), four had PapIva (CIN3) and one had PapIvb (⩾CIN3). The one woman with invasive carcinoma had a Pap smear classified as PapIII (ASC-H/AGUS). Human papillomavirus testing of these 37 women identified 36 women who were positive for HR-HPV, with the one case missed having a borderline negative result (0.83) that stayed negative after three-fold repeated retesting (average=0.993) with HC2 and was subsequently found to be positive for HPV 18 upon testing by PCR and sequence analysis. Of the 17 women who would have been detected by cytology, 16 were also positive for HR-HPV with the one discrepant case being the woman with CIN3 who was positive for HPV 18 on PCR/sequencing analysis. Using a case definition of CIN3+, the sensitivity, specificity, PPV and NPV of cytology were 46, 98.0, 9.7 and 99.7%, respectively, while the corresponding parameters for HPV testing were 97.3, 95.2, 8.7 and 100.0% (Table 3).

The sensitivity of cytology given above is based upon a Pap-smear result of ⩾PapIIw (≈borderline/ASCUS), which is lower than the usual threshold used in Germany. Using the usual threshold of ⩾PapIII (⩾ASC-H/AGUS), as the basis for referral to colposcopy, the sensitivity of cytology would have decreased to 37.0%, while the positive predictive value would have increased to 27.0% (Table 3). It has to be noted that the current analyses on test performances are based on the 7908 women with complete baseline and follow-up results. If we would include the women with a positive test for at least one of the tests at baseline that refused colposcopy (and were therefore excluded from the primary analysis) under the assumption that no cases would have been detected in these women, this leads to similar results as presented. Assuming, however, the other extreme that all 175 women who refused colposcopy would have been cases leads to a considerable lower sensitivity of the HPV test of 68.8%, while the sensitivity of cytology (43.0%) does not change significantly. Since the distribution of cytology or HPV positivity among the 175 women who refused colposcopy did not differ from the 711 participants who were examined, it is not likely that the exclusion of noncompliant women had any significant effect on the analysis.

A total of 925 of the routine cytology samples were reviewed by an independent expert, who noted at least minor deficiencies of sample fixation, staining or mounting in 19.8% of all slides. The primary cytology result and the review result were in agreement for 719 out of 925 (77.7%) Pap smears. In total, 87 out of 206 (42%) of the different cases involved a discrepancy of two or more grades (kappa value=0.25). Using review cytology with PapIIw (≈borderline/ASCUS) or higher as the basis for referral to colposcopy would have missed two cases identified on primary cytology, and would have identified an additional five cases.

QA testing of HC2-positive samples demonstrated a very high correlation between the two laboratories involved in this study. On the whole, 300 samples from the clinical laboratory at the Medizinische Hochschule Hannover were retested at the Department of Virology University of Tuebingen, and 300 samples from Tuebingen were retested in Hannover. In all, 96.6% of the samples yielded the same result on repeat HC2 testing for a kappa value of 0.75 (95% CI: 0.64–0.85).

## DISCUSSION

This trial is one of the first large-scale evaluations of HPV testing within an established nationwide cervical cancer-screening programme under routine screening conditions. Owing to the nonselected study population and the inclusion of rural and urban regions from both Northern and Southern Germany, the data are highly representative of Western Germany as a whole.

Ideally, routine screening for cervical cancer based on Pap smears should detect all high-grade neoplasia in a given population, while testing for HPV, as the causative agent of cervical cancer, should identify all individuals either having prevalent cervical neoplasia or of being at risk for developing incident cancer within the next decade. Combining both methods should enable a reliable risk stratification of the screened population and allow for the development of a risk-adapted screening programme with increased intervals for women at greatly reduced risk. Important aspects of this hypothesis are indeed supported by the results of this study. As expected, the group of Cyto+/HPV+ women contained the highest proportion of high-grade cervical neoplasia (19 out of 54), while no cases were found on colposcopic examination of 250 randomly selected women who were negative on both cytology and HPV testing. It is a limitation of this study that we were not able to examine 10% of double negatives as claimed by others to be adequate to assess verification bias, although the results from our colposcopy review process and preliminary follow-up data make it unlikely that cases were missed. This verifies the widespread belief that Cyto−/HPV− women are at significantly reduced risk of having high-grade CIN or cancer at the time they are tested. In addition, they should be protected for an extended period against the development of cervical cancer as suggested by other investigators (Rozendaal *et al*, 2000; Clavel *et al*, 2001). However, sufficient data are not yet available to define the length of the protective effect in Cyto−/HPV− women. Once defined, this could have an enormous impact on the frequency of testing and the cost efficiency of a screening programme. It should be noted, however, that lack of compliance for follow-up examinations as observed in this study among 20% of women being positive by either test compromises the success of any screening algorithm, independent of individual test performances.

The largest group of 26 cases was found among 460 women with normal cytology but positive HPV-test results, whereas only a single case was detected among 190 participants with abnormal cytology but negative HPV results. This high number of false-negative Pap-smear results could be because of the fact that routine standard Pap-smear procedures have been performed at office-based gynaecologists and cytology slides were read in private laboratories as part of the German cervical cancer-screening programme, which has considerably reduced the reimbursement rates for a Pap smear during the last decades. Other screening trials using a defined team of specialists for collecting samples and reading Pap smears indeed found a higher sensitivity for cytology for high-grade cervical neoplasia (Schiffman *et al*, 2000; Clavel *et al*, 2001) than observed here or in recent studies that evaluated routine Pap smears (Ratnam *et al*, 2000; Schneider *et al*, 2000). In addition, the standard recommended practice in Germany of taking samples with cotton-tipped swabs (Link M and Link H, 2001), which has been demonstrated to harvest fewer cells than other collection devices may also be responsible for these false-negative results (Martin-Hirsch *et al*, 1999) and may have biased the study results in favour of HPV testing.

A major factor contributing to the high number of ‘false-positive’ Pap smears was the cytological diagnosis ‘Pap IIw’ that denotes inflammation, minimal cytological changes or ASCUS, which is not part of the official Munich cytology classification system (Table 1). This diagnosis was the most frequent abnormal result with a total of 167 women (2.1%) receiving it. Only three cases of CIN2+ were found in women with PapIIw (≈borderline/ASCUS) results and all were associated with a positive HC2 result, while no case was detected among 149 (89.2%) women with PapIIw (≈borderline/ASCUS) and a negative HPV test result (Figure 2). In contrast, only one of 41 HPV− Pap smears either classified as PapIII (ASC-H) or as PapIIID (CIN 1 or CIN 2) was associated with high-grade disease. HPV testing proved to be highly efficient in triaging women with abnormal Pap findings because 19 out of 20 cases of underlying CIN2+ were HPV+, while 190 out of 251 patients with negative HC2 results could have been returned to the routine screening pool.

Considering the unsatisfactory performance of a single routine Pap smear seen in our trial and a former study from Germany (Schneider *et al*, 2000), it is questionable if a risk-adapted screening programme would benefit from the inclusion of conventional cytology. Whereas a negative HPV test result, even in combination with a positive Pap result, virtually excluded any risk of the underlying disease, a single negative Pap smear missed more than 50% of cases. Owing to this, cervical cancer screening in Germany relies on annual screening to achieve a sufficient sensitivity by repeated rounds of testing. This will, however, only work if the compliance among women participating in screening stays constant from year to year. In contrast, a screening protocol based on HPV testing alone would have detected 45 out of 46 cases in this study with a colposcopy referral rate of 11.6 (521/45) patients per case detected, while cytology detected only 20 out of 46 cases with a referral rate to colposcopy of 12.6 (251/20) patients per case detected. It has to be noted, however, that any effort to intensify cervical cancer screening, which increases the number of test-positive women, bears in conjunction with the low reproducibility of cervical cytology and histology observed in this study a potential risk of patient overexamination.

Although the high sensitivity and the high negative predictive value are obvious advantages of the HPV test, its use as a primary screening tool is hampered by its low specificity and the risk of possible overtreatment of HPV-test-positive women. One contributing factor to the low specificity is the crossreactivity of the HC2 high-risk hybridisation probe with low-risk HPV types or HPV types of undetermined risk as discussed in earlier studies (Peyton *et al*, 1998; Vernon *et al*, 2000; Solomon *et al*, 2001). Here we found a substantial degree of crossreactivity (6.4%) between the HC2 high-risk probe and HPV types not included in the probe cocktail by retesting all HC2+ samples with PCR followed by direct sequencing and by additional testing with the PGMY09/11-PCR system coupled to a reverse hybridisation line-blot assay (Kornegay *et al*, 2001) to be able to detect multiple infections. If the problem of crossreactivity theoretically could be eliminated, this would have increased the specificity of the HC2 test up to 97.6% without losing sensitivity (data not shown). In addition, we found a significant false-positive rate of 3.6% and a false-negative rate of 6.1% of all samples as defined by different results from HC2 *vs* a highly sensitive PCR/sequencing assay. Clearly, attempts to reduce or eliminate crossreactions and false positives/negatives would be a worthwhile strategy to achieve a better specificity of HPV testing, without affecting its sensitivity.

The use of an improved highly specific HPV test for primary cervical cancer screening in a generally low-risk population (6.4% HPV prevalence), as in Germany, holds great promise. Given the low prevalence of HPV infection in the female population aged 30 or above, combined with the high sensitivity of the HC2 test, ≈94% of women could have been returned safely to routine screening, while detecting virtually all of clinically relevant diseases. Theoretically, in a country like Germany, a significant benefit could be expected even from a twice in a lifetime HPV test. The timing of the first lifetime test should be sufficiently late in life, such that most HPV exposure has already occurred, but, on the other hand, sufficiently early to keep the risk of missing prevalent invasive cervical cancer to a minimum. It is expected that the cost efficiency of HPV testing will be poor in age groups under 30 years of age, because transient HPV infections are common (Evander *et al*, 1995) and cervical cancer is rare. The second lifetime test should be used to define type-specific persistence of an infection and include viral load analysis (van Duin *et al*, 2002), which is not yet available as a commercial routine test. Based on these results, suitable surveillance mechanisms, like colposcopy/biopsy, will have to be applied to follow-up patients with a substantially increased risk for cervical cancer. Further, if the preliminary results regarding the negative predictive value of HPV testing are confirmed in large-population-based studies over the longer term (>5 years), those women with low-risk sexual behaviour who are negative both on cytology and on HPV test could be grouped safely on a recall interval of 3–5 years or more, which would generate savings that could be applied to a better surveillance for those women who are at increased risk.
